# Origin Identification of Hungarian Honey Using Melissopalynology, Physicochemical Analysis, and Near Infrared Spectroscopy

**DOI:** 10.3390/molecules26237274

**Published:** 2021-11-30

**Authors:** Zsanett Bodor, Zoltan Kovacs, Csilla Benedek, Géza Hitka, Hermann Behling

**Affiliations:** 1 Department of Measurements and Process Control, Institute of Food Science and Technology, Hungarian University of Agriculture and Life Sciences, 14-16 Somlói Street, H-1118 Budapest, Hungary; Zsanett.bodor93@gmail.com; 2 Department of Dietetics and Nutrition, Faculty of Health Sciences, Semmelweis University, 17 Vas Street, H-1088 Budapest, Hungary; benedek.csilla@se-etk.hu; 3 Department of Postharvest, Commerce, Supply Chain and Sensory Science, Institute of Food Science and Technology, Hungarian University of Agriculture and Life Sciences, 43-45 D. Ménesi Street, H-1118 Budapest, Hungary; hitka.geza@uni-mate.hu; 4 Department of Palynology and Climate Dynamics, Albrecht-von-Haller Institute for Plant Sciences, University of Göttingen, 37073 Göttingen, Germany; Hermann.Behling@biologie.uni-goettingen.de

**Keywords:** honey, melissopalynology, origin, chemometrics, authenticity, data fusion

## Abstract

The objective of the study was to check the authenticity of Hungarian honey using physicochemical analysis, near infrared spectroscopy, and melissopalynology. In the study, 87 samples from different botanical origins such as acacia, bastard indigo, rape, sunflower, linden, honeydew, milkweed, and sweet chestnut were collected. The samples were analyzed by physicochemical methods (pH, electrical conductivity, and moisture), melissopalynology (300 pollen grains counted), and near infrared spectroscopy (NIRS:740–1700 nm). During the evaluation of the data PCA-LDA models were built for the classification of different botanical and geographical origins, using the methods separately, and in combination (low-level data fusion). PC number optimization and external validation were applied for all the models. Botanical origin classification models were >90% and >55% accurate in the case of the pollen and NIR methods. Improved results were obtained with the combination of the physicochemical, melissopalynology, and NIRS techniques, which provided >99% and >81% accuracy for botanical and geographical origin classification models, respectively. The combination of these methods could be a promising tool for origin identification of honey.

## 1. Introduction

Honey is a natural food and sweetener produced by honeybees (*Apis mellifera*) from the nectar and secretions of living parts of the plans, or also from excretions of sucking insect living on trees, this latter being called honeydew [1,2]. It is a source of several nutrients, such as sugars, organic acids, enzymes, minerals, vitamins, amino acids [3]. Depending on the botanical and geographical origin, the physico-chemical composition and sensory properties of honeys are different. The botanical origin covers the dominant plant source from which the honey is produced, while the geographical origin refers to the region from which the product is collected [4]. Only within Europe more than 100 species of plants are known to be sources of unifloral honeys; however, their frequency varies from rare to abundant [5]. In Hungary the most commonly sold honeys are acacia (*Robinia pseudocacia*), chestnut (*Castanea sativa),* rape (*Brassica napus*), sunflower (*Helianthus annuus*), linden (*Tilia* spp.), bastard indigo (*Amorpha fruticosa*), and silkweed (*Asclepias syriaca*) honeys. There are also other types sold, such as honeydew (from pine or other trees), phacelia (*Phacelia tanacetifolia),* and others. Hungary is one of the most important honey producers in the world and in Europe: with 21,000 tons Hungary was the 6th biggest honey producer of Europe in 2018. Origin identification of the honey is not easy as there is no reference honey [3,6] for the individual unifloral types due to their variability affected by different factors. These factors are processing technology, storage conditions, and the geographical origin (soil, climate, surrounding flora) [7]. The identification of the botanical origin according to the classical approach consists of three main sections: sensory analysis, determination of physicochemical properties, and melissopalynological analysis [8].

The melissopalynological examination is the identification of pollens in honey by microscopic examination, for which pollen reference collections are needed. “Unifloral honey” does not denote a honey containing exclusively a single type of pollen, but rather the dominance of pollen collected from a certain plant [9]. However, during pollen analysis it has to be taken into consideration that some plants can be good nectar producers, but low pollen producers. Thus, the pollen of such plants could be under-represented, such as in the case of the *Robinia pseudoacia* or citrus, while some types are normally or over-represented, such as *Brassica napus* or *Castanea sativa* pollen [10,11]. There is no general legal requirement for the unifloral honeys to contain an exact amount of pollen. Moreover, in different countries different rules apply for the different unifloral honeys [12]. In Hungary, for example, there is a special standard defining some requirements regarding the composition, sensory parameters, and pollen content of honeys: acacia honey should contain at least 15% *Robinia pseudoacacia* pollen, and linden honey at least 30% *Tilia* spp. pollen [13].

Determination of the chemical composition of honey requires different classical analytical methods [8,14,15,16,17]. Novel, so-called correlative analytical methods are often applied in honey authenticity evaluations. In the case of correlative techniques, the instrument does not directly measure certain components of the sample, but gives a fingerprint-like characterization of the sample. The results of different sample groups can be compared using different statistical methods and chemometrics. As a promising correlative method, near infrared spectroscopy (NIRS) has been applied for determination of the authenticity of different food products, including honey [18]. NIRS was successfully employed for the botanical origin identification of honey, resulting in 85.3% accuracy in a Chinese study [19]. In one of our previous study NIR spectroscopy was applied for the botanical origin identification, where handheld spectrometer provided 92% accuracy for the botanical origin and 95% for the geographical origin identification [20]. Nowadays it is possible to collect global information about the analytes using several multisensory/multivariate techniques even simultaneously, which can supplement each other, such as the NIRS, electronic nose, or electronic tongue [18]. The combination of the data (fusion) of different analytical techniques can provide better models in several cases [21,22,23,24] and has already been used in honey evaluation as well [21]. There are different types of data fusion techniques, such as low-level data fusion, when the different data sources are concatenated and the variables or the sets of variables (blocks) are weighed according to their importance [23]. In case of the mid-level data fusion the important features of the different datasets are combined and used for the further analysis. The feature determination can be done using dimension reduction models, such as principal component analysis or partial least square regression. Lastly, in high-level data fusion, which is also called decision level data fusion, the results of built models are combined and used for the data analysis. All the data fusion types have their own advantages and challenges [25,26]. There is no available literature on the data fusion approach of NIRS, physicochemical data, and melissopalynology. Therefore, these methods have never been applied in a combined way for the origin identification of honey.

The objective of this study is to determine the pollen spectra of some major types of unifloral honey samples from Hungary and to determine the applicability of the pollen analysis for the botanical and geographical origin identification. The study also aims to investigate the performance of the combined NIRS, pollen, and physicochemical analysis in the determination of the botanical and geographical origin of honey.

## 2. Results and Discussion

### 2.1. Results of the Physicochemical Analysis

Physicochemical analysis of the honey samples showed moisture content lower than 20% for all the honey samples, which fulfills the requirements of the legislations [1]. MANOVA analysis showed that the botanical origin affects significantly the physicochemical composition (moisture, pH, and electrical conductivity) of the honey samples (Table 1). However, the analysis of the individual parameters showed a significant effect of pH and electrical conductivity, but no significant effect was found for the moisture content. The electrical conductivity of the honey samples varied according to the botanical origin, but there was a high variability even between the honey samples from the same unifloral origin. The results obtained for the acacia, rape, linden, and sunflower honeys were in accordance with the results of a study investigating the composition of the main unifloral types in Europe [11]. The bastard indigo and milkweed honeys are not studied in depth in the literature, even though they are quite frequent in Hungary. Our results of pH and electrical conductivity were similar to the results of Chinese researchers [27] in the case of the bastard indigo honeys. Another Hungarian study on milkweed honeys found that the milkweed samples were more acidic than acacia honeys, as it was also proven by our study [28]. Chestnut honeys showed lower electrical conductivity in average than in the aforementioned European honey study, and in the case of some honey samples the values were below the limit of the European legislation (0.8 mS/cm). The same results were found in the case of the honeydew samples, where the average electrical conductivity was below the 0.8 mS/cm limit [1,11]. This could be due to some mislabeling, throwing light on a country-specific denomination problem: the term “*Erdei*” (meaning forest) is commonly used for honeydew honeys instead of the honeydew (“*Mézharmat*”) expression; however, “forest” honeys are mainly pollen-containing honeys, therefore this can lead to confusion.

### 2.2. Results of Melissopalinology

Upon the analysis of 87 samples, 107 different taxa were identified in total. The diversity of pollens in the different samples was influenced by the origin of the honeys. Rape and chestnut honey were characterized by lower pollen diversity compared to the others. Moreover, the geographical origin also had an effect on the pollen diversity of the samples. The concentration of the characteristic pollen of the different unifloral honeys was the following: in acacia honey the amount of *Robinina pseudacacia* pollen ranged between 7–43%. These results are in line with a Hungarian investigation analyzing Hungarian acacia honeys [29]. However, the requirement of the Hungarian norm for unifloral honeys was not met in the case of some acacia samples as the quantity of *Robinia pseudoacacia* pollen was lower than 15% [13]. In the case of linden honeys, *Tilia* pollen was found in the linden honey samples at 5.3–66.4%. Three of the samples contained less *Tilia* pollen than the 30% limit mentioned in the Hungarian standard [13]. There are no pollen requirements set for the other honey types in Hungary. The chestnut honeys contained *Castanea sativa* pollen in the range of 60–93%, rape honeys contained Brassicaceae (medium size 25–50 µm) pollen in the range of 15–91%. These two types are usually over-represented, therefore they should be present in higher amounts: according to the Hungarian standard for microscopical analysis of honey, chestnut and rape honeys should have at least 86% and 60% of the characteristic pollen, respectively. In the case of sunflower honey 3–83% of *Helianthus annuus* pollen was found in the unifloral types, while according to the Hungarian standard the *Helianthus annuus* pollen is sometimes under-represented (12–92%) [10,11]. Bastard indigo honeys contained *Amorpha fruticosa* pollen in the range of 13–94%. Unfortunately, there is no Hungarian or international requirement regarding this type of honey, but in a Chinese study 58% to 73% of *Amorpha fruticosa* pollen was found in the bastard indigo honeys [27]. In the case of the milkweed and honeydew honeys, there is no characteristic pollen, as *Asclepias syriaca* pollen is not present in the honey, while in the case of honeydew there is no characteristic plant source [10,11]. Therefore, for the following botanical model evaluations these samples were excluded from the analysis. The most important pollen types of the honeydew and milkweed honeys can be seen in the Table S1. Both honey types could be characterized by high pollen diversity (in honeydew 20–40 pollen taxa, in milkweed 19–32 pollen taxa were identified) and only in two of the samples were predominant pollen (present in >45%) taxa detected. Secondary pollen types (15–45%) were found in all of the samples: in the case of the honeydew Brassicacae, Apiaceae, *Amorpha fruticosa,* and Asteraceae were the most common pollens. In milkweed honey samples Papaveraceae and *Phacelia tanacetifolia* were found most frequently.

#### 2.2.1. Results of the Cluster Analysis of the Melissopalynological Data

**Figure 1:**
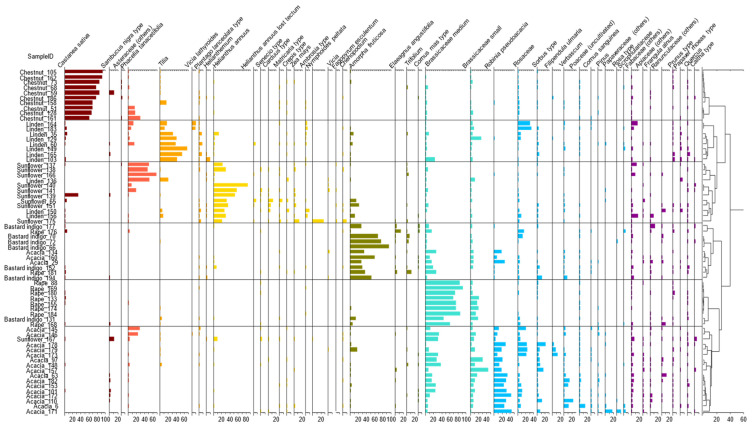
Pollen diagram of the main unifloral honeys after excluding taxa that present less than 2% frequency and the results of the cluster analysis.

Cluster analysis showed (Figure 1) that the whole dataset could be divided in two main groups: the first group contained chestnut, sunflower, and linden honeys, and the second group consisted of rape, acacia, and bastard indigo honeys. Honey samples belonging to the second group are usually characterized by similar composition as acacia and rape, which are usually low in minerals and phenolic compounds [11]. The first main group can be divided into the group of chestnut, sunflower, and linden honey subgroups. Chestnut samples were belonging to one big subgroup, but a lower separation level was found, where three samples were separated to another subgroup within the chestnut cluster. Those samples were lower in *Castanea sativa* pollen and contained *Phacelia tanacetifolia* pollen in higher amounts. This can be due to the collection time of the pollen, as chestnut in Hungary flowers from May to early July, but *Phacelia* flowers merely during the entire summer, even in early July and late July [30]. Therefore, those samples could originate from a later harvest. *Phacelia tanacetifolia* pollen was found in higher amount in sunflower honey and in linden honey. This can be traced back to the fact that *Phacelia tanacetifolia* is the intermediary nectar source for bees between the collection period of the acacia and sunflower; however, its presence highly depends on the sowing time. The cluster is composed of linden honey samples, although three linden honeys were clustered together with the sunflower honeys. Besides *Tilia*, in linden honeys Rosaceae, Brassicaceae, *Plantago lanceolata, Vicia lathyroides,* and *Frangula alnus* pollens were found in higher amounts. The three linden honeys grouped with the sunflower honeys also showed higher *Helianthus annuus* and/or *Phacelia tanacetifolia*, and less *Tilia* pollen contents compared to the other linden honeys. This can also be due to the later collection of linden honey as the *Tilia* plants mainly bloom in June, but sunflower starts flowering in the end of June and flowers during July. The higher *Phacelia tanacetifolia* pollen content in those samples could also be due to the fact that *Phacelia* is usually an over-represented pollen type, and bees prefer to collect them [30]. The other big group consists of acacia, rape, and bastard indigo honeys. One subcluster is mainly composed of bastard indigo honeys, while also some acacia and a rape honeys were included here. The bastard indigo samples contained Brassicaceae, *Salix*, *Trifolium*, and *Elaegnus* pollen. This latter was specific for this honey type. Rape samples that belong to the bastard indigo group were richer in *Amorpha fruticosa* pollen than the other samples from this type; however, it is also worth mentioning that all the samples originated from the region of Tisza river (that flows through the Great Plain), where the bastard indigo plants are the most common in Hungary [31]. Another subcluster includes rape honeys, which were rich in Brassicaceae pollen, but *Salix* was also found in them. One of the bastard indigo samples was clustered together with the rape honeys that contained more Brassicaceae pollen and low amounts of *Amorpha fruticosa* pollen. The last bigger cluster subgroup consisted mainly of acacia samples, but one sunflower honey—which was low in sunflower pollen—was also clustered to the acacia honeys. Acacia honey samples mainly contained the pollen of the *Robinia pseudoacacia*, Brassicacea, Rosaceae, and *Verbascum* plants.

#### 2.2.2. Results of the Botanical Origin Identification Models Using the Pollen Data

**Figure 2:**
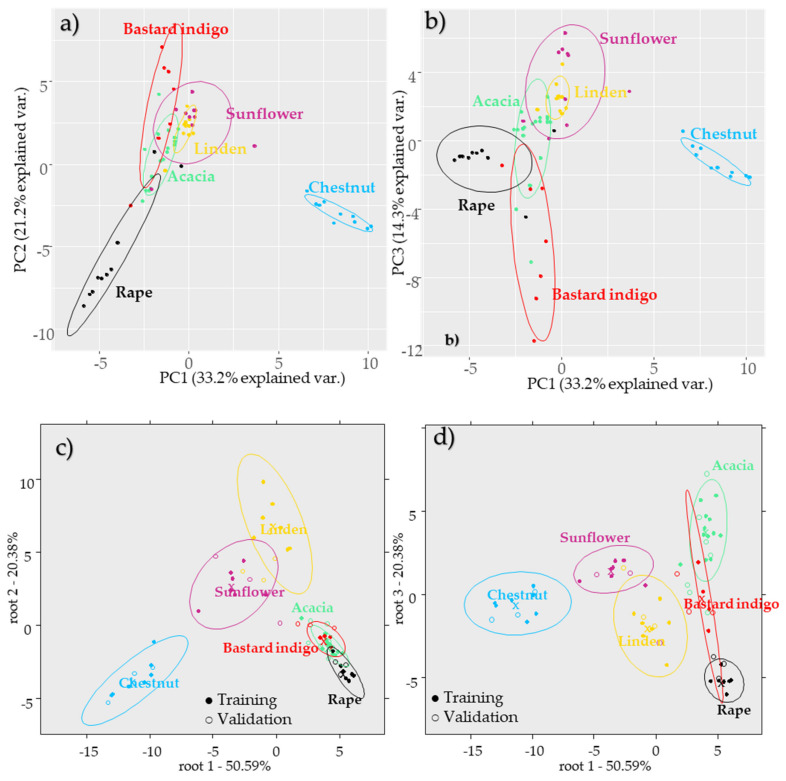
Multivariate models of the honey samples according to botanical origin based on the pollen data. Principal component analysis score plot (**a**) PC1-PC2, (**b**) PC1-PC3; PCA-LDA score plot using 17 PCs, (**c**) discriminant functions: root 1-root 2, and (**d**) discriminant functions: root 1-root 3.

Principal component analysis of the honey samples (Figure 2) using the pollen data (excluding the taxa represented under 2%) showed a separation tendency according to the different unifloral types. The first principal component PC-1 described 33.2% of the variance; separation of the chestnut honey samples from the other types (Figure 2a) was observed through this PC. *Castanea sativa*, Asteraceae, and *Sambucus nigra* type pollens contributed mostly to the separation of the chestnut honeys. Along PC-2 the separation of rape honey was observed. PC-2 described 21.2% of the total variance, where the Brassicaceae medium, Brassicaceae small, and *Prunus* type contributed mostly to the separation of the rape samples. PC-3 described 14.3% of the total variance, where the separation of the linden, sunflower, and acacia samples can be seen from the other honey types (Figure 2b).

The separation of bastard indigo samples can be attributed to the presence of *Amorpha fruticosa, Cornus mas, Trifolium, Elaeagnus angustifolia,* and *Frangula alnus* pollens. This was also seen on the pollen diagram (Figure 1). The presence of *Robinia pseudoacacia* and Poaceae contributed to separation of the acacia samples, while *Matricaria* type, *Crepis* type, *Achillea* type, *Helianthus annuus,* and *Phacelia tanacetifolia* pollens contributed to the separation of sunflower honey. However, linden and sunflower honeys did not show a neat separation tendency (Figure 2b), that was also seen on the pollen diagram, where these clusters were close and some of linden samples were clustered together with the sunflower honey samples (Figure 1).

PCA-LDA analysis showed also a good separation of the groups according to the botanical type. According to the plots (Figure 2c,d) the rape honey was completely separated from the rest of the sample groups; however, the sunflower and linden honeys showed an overlapping with each other, and so did the acacia, rape, and bastard indigo groups. However, these three showed a separation tendency alongside root 3. After the external validation the classification models provided the average recognition (training set) and prediction abilities (external validation set) of 98.61% and 91.67%, respectively. During the training all the groups were classified correctly with the exception of acacia, where misclassification was found as belonging to the bastard indigo group in 8.33%. Validation set showed 100% classification accuracy for rape, acacia, sunflower, bastard indigo, and chestnut groups. In the case of linden misclassification was found as belonging to the sunflower group in 50%. The misclassifications can be explained by the fact that some of the linden honeys contained higher amounts of sunflower pollen, and in some acacia honeys also higher rate of bastard indigo pollen was detected (Figure 1).

PCA-LDAs of the pollen data (using 9 PCs) built for the classification according to geographical regions resulted in average recognition (training set) and prediction (external validation set) abilities of 64.42% and 58.08%, respectively. These results showed that the effect of the botanical origin is higher than that of the geographical origin. All the groups showed misclassifications as belonging to other regions. The detailed results can be seen in Table S2.

### 2.3. Results of the near Infrared Spectroscopy

**Figure 3:**
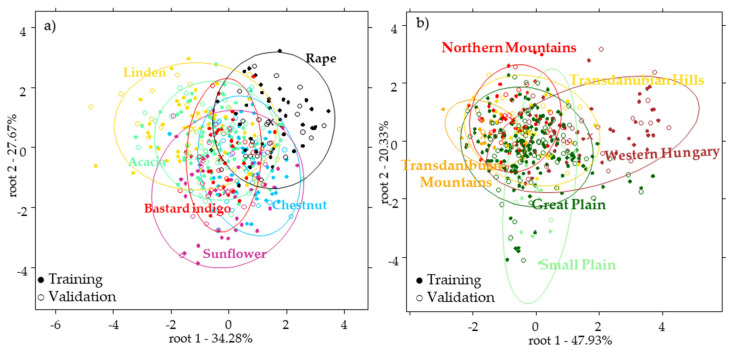
Results of the PCA-LDA models built using the NIR data for the classification of honey for the (**a**) botanical origin discriminant functions: root1-root2 using 28 PCs and (**b**) geographical origin discriminant functions: root1-root2 using 18 PCs.

NIRS results were analyzed using different pretreatment combinations from which the combination of Savitzky-Golay smoothing (2nd polynom, 21 filter length) + Savitzky-Golay smoothing (2nd polynom, 21 filter length, 1st derivative) provided the best results for the classification of the botanical origins. Therefore, the models were built using this pretreatment combination. The PCA results showed that the first four principal components described 99% of the variance; however, no clear separation was observed according to the botanical groups. PCA-LDA models also showed overlapping among the groups and the average recognition (training set) and prediction abilities (external validation set) were 61.35% and 55.34%, respectively (Figure 3a). The most accurate classification (based on the external validation set) was obtained for the rape honeys (66.67%), followed by acacia (64.29%), linden (52.00%), chestnut (52.00%), sunflower (50%), and bastard indigo (47.06%). These results show that for this sample set it was not possible to achieve acceptable classification accuracy using NIRS (Figure 3a). The reason of this relatively weak classification accuracy could be due to the lack of the spectral region of the bands associated with sugars which is above 1650 nm [32]. Thus, the important differences between the sugar composition of the different unifloral samples that could be reflected in the spectra, and thus provide better classification, were not measured.

Results of the PCA-LDA model for the classification of the geographical regions provided worse results compared to the botanical model such as in the case of the models of the pollen data. The average training and external validation accuracies (were 43.06% and 40.95%, respectively. However, a separation tendency can be seen (Figure 3b), where the honeys originating from higher altitudes are placed on the top part, while honeys from the plains can be found in the bottom part of the plot. None of the groups was classified correctly, the classification accuracies are shown in the Table S3. Similarly to the pollen models, these results prove the higher effect of the botanical origin.

### 2.4. Results of the Combined Data

The PCA results of the combined data of the pollen, NIRS, and physicochemical analysis showed a nice separation after standardization according to the botanical origin, mainly through the PC1-PC4 pair. The first four principal components described 88.5% of the total variance. The separation according to the geographical origin was not that obvious.

**Figure 4:**
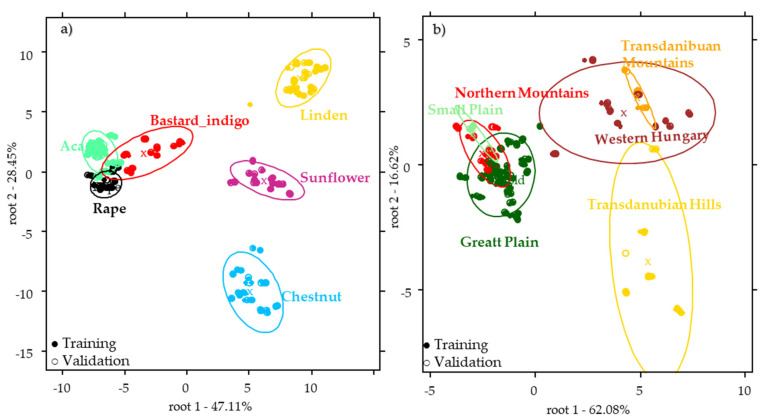
Results of the PCA-LDA model of honeys built using the fused data of pollen, NIR, and physicochemical analysis for the classification of the (**a**) botanical origin discriminant functions: root1-root2 using 29 PCs and (**b**) geographical origin disriminant functions: root1-root2 using 28 PCs.

PCA-LDA results of the merged dataset showed improved classification accuracies in the case of botanical and geographical models, too. PCA-LDA model of the botanical origin provided 99.35% and 99.21% average classification accuracies of the training and external validation data set. All the sample groups were classified correctly with the exception of acacia, where the samples were misclassified as belonging to the bastard indigo group in 3.9% and 4.76% during the training and validation, respectively. This model is better than the models obtained for the pollen or NIR data separately. The misclassified sample points belonged to one sample that was the Acacia_160. This sample was also clustered together with the bastard indigo samples when analyzing only the pollen data. The sample contained only 7% of *Robinia pseudoacacia* pollen, but 59.7% of *Amorpha fruticosa*. Therefore, in the case of this honey a mislabeling is supposed. The reason for this could be that this sample originates from the region of Tisza river, where the bastard indigo plants are quite frequent and their efflorescence periods are overlapping with those of acacia. Based on the biplot (Figure 4a), it can be seen that the groups of acacia, rape, and bastard indigo samples were close to each other. The physicochemical composition of these samples has been found to be similar, and no significant difference was found between these honey types (Table 1). The variables that mostly contributed to the distinction of these three groups were the Brassicaceae (medium size), Brassicaceae (small size), *Pinus, Amorpha fruticosa,* and *Robinia pseudoacacia* pollens. Sunflower and linden honeys were also closer to each other (through root 1) than to other sample groups. However, linden and sunflower honeys separated clearly through the root 2, where the *Tilia, Papaver rhoeas, Plantago lanceolata,* Fabaceae (others), *Ambrosia* type, *Vicia lathyroides,* and *Caltha* type pollens contributed to the separation of the linden, while *Phacelia tanacetifolia, Carduus* type, and *Helianthus annuus* pollens and pH had the highest role of the distinction of the sunflower honeys. Chestnut samples were separated completely from all the other sample groups, where the highly contributing variables were the electrical conductivity and the presence of *Castanea sativa* pollen, based on the loadings of the model.

The PCA-LDA model using the fused data for the geographical origin identification provided 83.79% and 81.60% classification accuracy for all the sample groups, during the training and external validation. During the validation Small Plain was classified correctly, while the Great Plain showed 2.86% misclassification as belonging to the Northern Mountains and Small Plain (Table S4). Honeys from the Transdanubian Hills were misclassified as belonging to the Western Hungary (15.38% misclassification) and the Transdanubian Mountains (50% misclassification). The reason for this could be that these parts of the country are close to each other. Western Hungary showed 85.71% correct classification, the misclassified samples were classified as belonging to the Great Plain. This could be explained by the fact that the misclassified observations belonged to a rape honey (Rape_169). Most of the rape honeys originate from the Great Plain and the effect of botanical is still higher than that of the geographical. Figure 4b shows the score plot of the geographical model, where the samples originating from the western part of the country can be found on the right side of the plot, while groups from eastern part and the Small Plain are on the left side. In this case the *Tilia*, *Papaver rhoeas* type, *Vicia*, *Filipendula ulmaria, Vicia lathryroides* pollens, pH, moisture, and electrical conductivity contributed to the separation of the sample groups from Western Hungary and Transdanubia. *Filipendula ulmaria* mostly lives in the western part of Hungary, similarly to *Tilia*. Samples in which *Vicia lathyroides* pollen was found originated from these regions, and the samples containing higher % of *Filipendula ulmaria* pollen were collected from Western Hungary. As regards the distinction of the Great Plain, Small Plain and Northern Mountains, the *Cornus sanguinea*, *Ambrosia* type, *Helianthemum*, *Sambucus nigra* type, *Senecio* type, *Amorpha fruticosa*, and *Ranunculaceae* (others) pollens contributed with the highest loading. It can be also observed that most of these pollen types (Figure 1) were associated (during the pollen analysis) with the acacia, rape, bastard indigo, and sunflower honeys. Moreover, it should be noted that 18 acacia, 6 rape, all the bastard indigo, and all the sunflower honeys were collected in these regions.

## 3. Materials and Methods

### 3.1. Honey Samples

**Figure 5:**
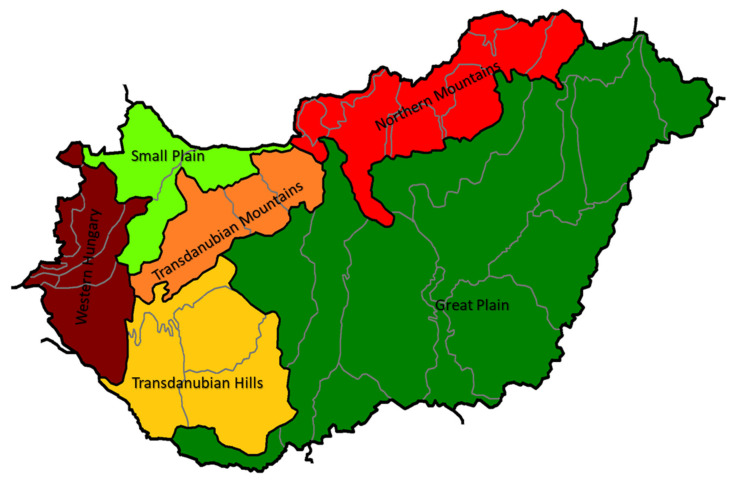
The geographical regions of Hungary where the tested honey samples were collected from.

Honey (*n* = 87) samples were collected directly from reliable beekeepers in the timeframe of 2015–2020. The samples were stored in a dark place at room temperature. The most common Hungarian honey types, such as acacia (*n* = 19), rape (*n* = 10), sunflower (*n* = 10), chestnut (*n* = 10), milkweed (*n* = 10), linden (*n* = 11), honeydew (*n* = 10), and bastard indigo (*n* = 7) samples were analyzed. The samples originated from different regions of Hungary (Table S5), such as Great Plain (Alföld), Northern Mountains (Északi- középhegység), Small Plain (Kisalföld), Transdanubian Hills (Dunántúli-dombság), Transdanubian Mountains (Dunántúli-középhegység), Western Hungary (Nyugat-magyarországi peremvidék) (Figure 5).

### 3.2. Determination of Physicochemical Parameters

Moisture content, electrical conductivity, and pH of the samples were determined according to the method guide of the International Honey Commission (Bogdanov, 2009). For moisture content two readings per sample, and for pH and electrical conductivity three readings per sample have been recorded. Moisture content analysis was performed using an Abbé-type refractometer (Carl Zeiss, Jena, Germany) and the results of the readings were adjusted to the refraction indexes of the above guide. The electrical conductivity and pH measurements were done using a Mettler Toledo SevenMulti analyzer (Mettler Toledo, Columbus, Ohio, USA). The pH was determined with a Mettler Toledo Inlab Expert Pro-ISM probe (Mettler Toledo, Columbus, OH, USA) and the electrical conductivity was measured with a Mettler Toledo Inlab731 probe (Mettler Toledo, Columbus, Ohio, USA).

### 3.3. Melissopalynology

Pollen analysis of the samples has been performed using the acetolysis method [33], similarly to the article published earlier [34]. First, 3–5 mL of honey was put in a centrifuge tube and filled up to 14 mL with distilled water. The samples were thoroughly mixed with the water and centrifuged at 3500 rpm for 5 min. After decanting the supernatant, this step was followed by the dehydration using acetic acid: 4 mL of acetic acid was added to the samples and mixed well, then centrifuged as previously. Supernatant was decanted and the acetolysis was performed: 4 mL of the solution of 1:9 sulfuric acid:acetic anhydride solution was added to each tube, then tubes were mixed well and put in water bath for 7 min at 90 °C. The samples were mixed again carefully and centrifuged. This step was followed by decantation of the supernatant and washing with 6 mL of distilled water. Samples were mixed well repeatedly and centrifuged. Then pollen from the bottom of the centrifuge tube was pipetted to Eppendorf tubes. The permanent pollen slides were prepared using gelatinized glycerin. All the samples were counted up to at least 300 pollen grains at 400× magnitude using light microscope (Carl Zeiss AG, Aalen, Germany). In cases where samples did not provide sufficient pollen, the same procedure was repeated using more honey. The pollen identification was performed using the reference collection of the Department of Palynology and Climate Dynamics of the University of Göttingen and an atlas of Central European pollen [35].

### 3.4. Near Infrared Spectroscopy

Near infrared spectra of the samples were recorded using a benchtop MetriNIR (MetriNIR Research, Development and Service Co., Budapest, Hungary) instrument in the spectral range of 740–1700 nm. The spectra were recorded in transflectance mode. The layer thickness of the samples in the cell was 0.5 mm, and the cell was thermo-regulated at 25.0 °C. Before the measurement samples were completely liquified at 37 °C to exclude the light scattering problems caused by the crystals in the sample. The samples were cooled down to room temperature before the spectral acquisition. Samples were filled into the thermo-regulated cell and the reflector was inserted carefully to make sure there are no bubbles formed in the honey layer, then after one minute temperature stabilization (25 °C) samples were measured three times. After this, the cell was turned by 90° and the same procedure was repeated twice, this resulting in a total of nine (3 position × 3 consecutive scans) scans per sample.

### 3.5. Statistical Analysis

#### 3.5.1. Physicochemical Data

Results of the physicochemical analysis were evaluated using descriptive statistics, where average and standard deviation were calculated. Significant differences between the sample groups were analyzed using multivariate variance analysis (MANOVA). The assumptions were tested: normal distribution of the results were analyzed using Shapiro-Wilk test, the homoscedasticity was tested using Box-test, homogeneity of the variances for the individual parameters was tested using Levene-test. In the case of significant MANOVA analysis we also tested for the individual parameters using Bonferroni correction. In the case of significant Levene-test (*p* < 0.05 homogeneity not assumed) the post hoc comparison was performed with Games-Howell test, as it is not sensitive for the inhomogeneity of the variances, in other cases Tukey-test was applied [36]. Data analysis was performed in SPSS 25 software (IBM SPSS Statistics, IBM, Endicott, New York USA).

#### 3.5.2. Pollen Data

Illustration of the pollen data was done using TILIA and TILIAGRAPH software [37], where pollen diagram was created from the pollen data excluding the pollen taxa that were found in amounts of less than 2% in the samples. These latter were probably not actively collected by the bees or were usually present in honey by mistake (like wind-pollinated species); therefore, they were omitted during the analysis. Percentages were used for the data interpretation in proportion to the total counted pollen grain per sample. Cluster analysis was calculated using total sum of scares method based on Edwards’ Cavalli-Sforza’s Chord distance. The cluster analysis was used with unconstrained method using CONISS [38]. The counted percentages were further processed in Excel, where principal component analysis (PCA) and PCA-linear discriminant analysis (PCA-LDA) [39] were performed on the pollen data using 95% confidence ellipses. The PCA-LDA models were validated using PC number optimization (with cross validation) and external validation. First the optimal number of the principal components (PCs) were determined on 2/3 of the data, then the remaining 1/3 data was used to test the accuracy of the model. The training set used for the determination of the optimal number of PCs contained the 2/3 of the data, in a balanced way, i.e., from each botanical/geographical group 2/3 of the observations were selected randomly. The PC number optimization was performed using different numbers of PCs (from 1–30) and using leave-one-out validation. Then the training and validation accuracies of the training set were compared. The PC number providing the smallest difference between the accuracies of model building and cross-validation and at the same time the highest validation accuracy was used to build the final model using 2/3 of the data used for the optimization (training set—recognition). In the secondary validation phase the remaining 1/3 of the observations were predicted into the model to perform external test set validation (prediction). Classification models were built for the discrimination of botanical and geographical origin, where in the case of the model bult for the classification of the geographical origin samples with non-confirmed origin were not included in the modeling.

#### 3.5.3. NIRS Data

NIRS data analysis was performed using R-project software and Aquap2 package [40]. Before data analysis using the raw spectra, outlier detection was performed using the built-in outlier detection function based on sample groups. Then 41 pretreatment combinations (Table S6) were applied to check the combination that provides the best model for the botanical origin classification, then in the following the best pretreatment was applied for data interpretation. The optimization of the NIRS-based PCA-LDA models was similar to the optimization of the models built using the pollen data. However, in this case the spectral pre-treatment optimization was also implemented before the determination of the optimal number of PCs. First the pre-treatment of the dataset was performed, then PC number optimization was used for the already pre-treated dataset. After each pretreatment the PC number optimization was performed the following way: as in the case of NIR measurements each sample was measured in three parallel sample replicates recording three consecutive scans on each, the 2/3 of the dataset was formed in a way that it included all the spectra of two parallel sample replicates. Then LDA models with different PC numbers (1–50) were built for the training set using leave-one-sample-out (including each of the three consecutive scans) cross validation. This was followed by comparison of the training and validation accuracies of the training set. The optimal PC number was chosen the same way as in the case of the pollen data evaluation. The PCA-LDA model using the chosen PC numbers were built for the training set (recognition) and in the secondary validation phase the other 1/3 of the observations were predicted into the model to perform external test set (prediction) validation. The PCA-LDA models were built for the classification of the botanical and geographical origin.

#### 3.5.4. Fusion of the Data

The fusion of the data was also created using the low-level fusion approach. The collected NIR spectra (after a pretreatment aiming for the best accuracy in the NIR modeling and outlier detection) were concatenated with the average value of the physicochemical parameters of the sample and with the melissopalynology percentages of the sample (after excluding pollens under 2%) (1st level preprocessing). Then scaling and mean-centering of the data were applied before model developments (2nd level preprocessing) [41]. Mean-centering was performed by subtracting the mean value of each variable (wavelengths or sensors) from the respected single values. Then, for scaling, the mean-centered values were divided by the standard deviation of the corresponding variable. The fused data set was analyzed with PCA-LDA analysis. The PC number optimization and external-cross validation was performed the same way as in the case of NIRS.

## 4. Conclusions

Herein, 87 Hungarian honey samples from different botanical (acacia, chestnut, rape, sunflower, milkweed, honeydew, linden, and bastard indigo) and geographical origins were analyzed using physicochemical data (pH, electrical conductivity, and moisture content), melissopalynological, and NIRS analysis. The results showed that the botanical origin has a pronounced effect on the physicochemical properties of the samples, where significant differences were found in pH and electrical conductivity among the unifloral types. Pollen analysis of the samples showed clearly separated clusters related to the botanical origins; however, some honeys were not clustered in their own group. Furthermore, melissopalynological data showed a good classification accuracy (>90%) when modeling the botanical origin discrimination; nevertheless, the model for the geographical origin performed in a weaker manner. The PCA-LDA model built for the NIRS data could not provide effective differentiation of the samples neither for botanical, nor for geographical origin. However, the combination of the three different analyses using low-level data fusion (that was applied the first time in honey analysis) showed high accuracy for the discrimination of the botanical >99% and geographical origins >80%. Our results proved that this method combination has a high potential not only in the origin identification of honey, but also in revealing typical mislabeling problems. However, authors suppose that the further optimization and application of other types of classification techniques and fusion approaches could further improve the models, which needs supplementary investigations in the future.

## Figures and Tables

**Table 1:** Results of the physicochemical parameters of the analyzed honey samples by botanical groups.

Botanical Origin	Moisture %	Electrical Conductivity µS/cm	pH
**Acacia**	17.7 ± 1 ^a^	148.1 ± 20.4 ^a^	4.0 ± 0.2 ^bcd^
**Bastard indigo**	17.6 ± 1.1 ^a^	305.3 ± 167.7 ^abc^	3.9 ± 0.3 ^abd^
**Chestnut**	16.8 ± 1.8 ^a^	715.1 ± 120.6 ^c^	4.4 ± 0.2 ^e^
**Honeydew**	17.3 ± 1.2 ^a^	566.1 ± 205.2 ^bc^	4.2 ± 0.2 ^cde^
**Linden**	17.7 ± 1.4 ^a^	617.6 ± 134.1 ^bc^	4.3 ± 0.3 ^ce^
**Rape**	18 ± 1.1 ^a^	231.9 ± 75.2 ^a^	4.0 ± 0.1 ^bcde^
**Milkweed**	18.1 ± 1.4 ^a^	264.2 ± 106.3 ^a^	3.8 ± 0.1 ^a^
**Sunflower**	17.4 ± 1.1 ^a^	472.6 ± 96.4 ^b^	3.8 ± 0.4 ^ab^

Mean ± Standard deviation. Letters denote the significant differences among the groups within each parameter based on the MANOVA analysis after pair-wised comparison at *p* < 0.05.

## Data Availability

The data presented in this study are available on request from the corresponding author. The data are not publicly available, due to privacy reasons.

## Supplementary Materials

The following are available online. Table S1: The most important pollen types of the honeydew and milkweed honeys; Table S2: Confusion matrix of the model of the pollen data for the classification of geographical origin; Table S3: Confusion matrix of the model of the NIR data for the classification of geographical origin; Table S4: Confusion matrix of the model of the fused data for the classification of geographical origin; Table S5: List of the analyzed samples and their botanical and geographical sources; Table S6: Pretreatments of the NIR spectra.

## Sample Availability

Samples of the compounds are not available from the authors.
