# Supplementary material for: Origin Identification of Hungarian Honey Using Melissopalynology, Physicochemical Analysis, and Near Infrared Spectroscopy
Source: Molecules. 2021 Nov 30;26(23):7274. doi: 10.3390/molecules26237274 (PMC8658813; doi:10.3390/molecules26237274)
Supplement: Supplementary file 1 [file molecules-26-07274-s001.zip › Table S1.pdf]

Table S1. The most important pollen types of the honeydew and milkweed honeys

Sample	Predominant pollen >45%	Secondary pollen 16-45%	Important minor pollen 4-15%
**Honeydew_48**		*Phacelia tanacetifolia - 32.67%,* Brassicaceae small *- 16.67 Helianthus annuus - 16%, %*	Brassicaceae medium - 13.67%
**Honeydew_55**		Ranunculaceae (others) *- 18.64%*	*Zea mays - 10.85%,* Brassicaceae medium - 8.81%, *Senecio type - 7.8%, Trifolium - 6.44%,* Rosaceae - 6.44%, *Tilia - 5.42%, Plantago lanceolata type - 5.08%,* Poaceae (uncultivated) - 4.75%
**Honeydew_62**		*Daucus/Bifora type - 37%, Amorpha fruticosa - 16.67%*	*Ambrosia type - 6.33%, Androsace - 6%, Artemisia - 6%*
**Honeydew_71**	*Solanum nigrum type - 60.33%*		Brassicaceae medium - 6.67%, Rosaceae - 5%
**Honeydew_85**		*Amorpha fruticosa - 27%,* Rosaceae - 16%, *Filipendula ulmaria - 15.33%*	*Sorbus type - 7%, Cornus sanguinea - 5.33%*
**Honeydew_130**		*Coriandrum sativum - 20.4%, Foeniculum vulgare - 16.05%,* Fabaceae (others) - 15.38%	Brassicaceae medium - 8.03%, *Ambrosia type - 6.35%, Carduus type - 6.35%,* Apiaceae (others) - 6.02%
**Honeydew_147**		*Amorpha fruticosa - 21.67%*	*Phacelia tanacetifolia - 10%,* Brassicaceae medium - 9%, *Helianthus annuus - 8.67%, Sorbus type - 5.67%, Castanea sativa - 4.33%,*
**Honeydew_170**		*Helianthus annuus - 16%,* Brassicaceae medium - 15.33%	Brassicaceae small - 7.33%, *Plantago major/media type - 7.33%, Carduus type - 6.33%, Chenopodium - 5.67%, Senecio type - 5.33%, , Artemisia - 4.33%,*
**Honeydew_188**		*Senecio type - 21.67%*	*Trifolium - 10.67%, Tilia - 10%, Plantago lanceolata type - 9.33%,* Rosaceae - 8.67%, Fabaceae (others) - 7%, *Phacelia tanacetifolia - 5.67%, Plantago major/media type - 4.67%*

Sample	Predominant pollen >45%	Secondary pollen 16-45%	Important minor pollen 4-15%
**Honeydew_189**		*Castanea sativa - 25.99%,* Brassicaceae medium - 19.74%,	*Quercus - 12.5%, Sorbus type - 6.58%, Ambrosia type - 5.26%,*
**Milkweed_32**		Papaveraceae (others) - 18.33%, *Trifolium - 17%*	*Caltha type - 11.33%, Tilia - 7.33%, Amorpha fruticosa - 6.33%, Plantago lanceolata type - 5.33%, Carduus type - 4.33%,* Brassicaceae medium - 4.33%,
**Milkweed_50**		*Tilia - 16%*	*Phacelia tanacetifolia - 13.67%, %, Papaveraceae (others) - 13.33%, Helianthemum - 10.33%, Amorpha fruticosa - 6.33%, Caltha type - 5.33%, Scrophulariaceae - 5%,* Brassicaceae small - 4.67%,
**Milkweed_52**		Papaveraceae (others) - 23.67%	*Helianthus annuus - 14.33%, Caltha type - 10.67%, Verbascum - 9%* Brassicaceae medium - 6.33%, Brassicaceae small - 6.33%, *Rosaceae - 4.33%*
**Milkweed_132**		*Allium vineale type - 37.33%*	Brassicaceae small - 9%, *Verbascum - 8.33%, Amorpha fruticosa - 6%, Robinia pseudoacacia - 4.67%,*
**Milkweed_185**		Papaveraceae (others) - 22.33%	*Helianthus annuus – 12, Plantago major-media type - 10.33%, %,* Brassicaceae medium - 8.33%, Brassicaceae small - 6.33%
**Milkweed_187**		Brassicaceae medium - 21%, *Phacelia tanacetifolia - 18%*	Brassicaceae small - 10.67%, *Robinia pseudoacacia - 9.33%, Verbascum - 6.33%, Quercus - 5.67%, Sorbus type - 5%*
**Milkweed_190**	*Phacelia tanacetifolia - 51.31%*	-	Brassicaceae small - 12.75%, Brassicaceae medium - 10.13%, *Castanea sativa - 6.54%, Sorbus type - 6.54%*

Sample	Predominant pollen >45%	Secondary pollen 16-45%	Important minor pollen 4-15%
**Milkweed_191**		*Phacelia tanacetifolia - 23.67%*	Brassicaceae small - 10.67%, *Amorpha fruticosa - 8.67%, Trifolium - 8.67%,* Brassicaceae medium - 7.67%, *Sorbus type - 5%Echium vulgare - 4.67%*
**Milkweed_192**		*Castanea sativa - 35%*	*Rhus typhina - 8.67%,* Brassicaceae small - 7.67%, *Echium vulgare - 4.67%*
**Milkweed_193**		*Sorbus type - 16%*	*Robinia pseudoacacia - 14.67%, Frangula alnus - 14%, Rosaceae - 13.33%,* Brassicaceae small - 7%, *Amorpha fruticosa - 5.33%*
