# Supplementary material for: Origin Identification of Hungarian Honey Using Melissopalynology, Physicochemical Analysis, and Near Infrared Spectroscopy
Source: Molecules. 2021 Nov 30;26(23):7274. doi: 10.3390/molecules26237274 (PMC8658813; doi:10.3390/molecules26237274)
Supplement: Supplementary file 1 [file molecules-26-07274-s001.zip › Table S2_mod.pdf]

Table S2 Confusion matrix of the model of the pollen data for the classification of geographical origin

		Great Plain	Transdanubian Hills	Transdanubian Mountains	Northern Mountains	Small Plain	Western Hungary
**Traning 64.42%**	**Great Plain**	**68.18**	0	0	40	25	16.67
	**Transdanubian Hills**	9.09	**75**	0	0	0	0
	**Transdanubian Mountains**	0	0	**50**	0	0	0
	**Northern Mountains**	13.64	0	0	**60**	25	0
	**Small Plain**	9.09	0	0	0	**50**	0
	**Western Hungary**	0	25	50	0	0	**83.33**
		**Great Plain**	**Transdanubian Hills**	**Transdanubian Mountains**	**Northern Mountains**	**Small Plain**	**Western Hungary**
**Validation 58.01%**	**Great Plain**	**81.82**	50	0	100	50	33.33
	**Transdanubian Hills**	0	**50**	0	0	0	0
	**Transdanubian Mountains**	0	0	**100**	0	0	0
	**Northern Mountains**	18.18	0	0	**0**	0	0
	**Small Plain**	0	0	0	0	**50**	0
	**Western Hungary**	0	0	0	0	0	**66.67**
