# Supplementary material for: Origin Identification of Hungarian Honey Using Melissopalynology, Physicochemical Analysis, and Near Infrared Spectroscopy
Source: Molecules. 2021 Nov 30;26(23):7274. doi: 10.3390/molecules26237274 (PMC8658813; doi:10.3390/molecules26237274)
Supplement: Supplementary file 1 [file molecules-26-07274-s001.zip › Table S3_mod.pdf]

Table S3 Confusion matrix of the model of the NIR data for the classification of geographical origin

		Great Plain	Transdanubian Hills	Transdanubian Mountains	Northern Mountains	Small Plain	Western Hungary
**Traning 43.06%**	**Great Plain**	**83.92**	60	45.45	68.42	53.85	48.78
	**Transdanubian Hills**	4.2	**32**	9.09	2.63	0	2.44
	**Transdanubian Mountains**	0	0	**36.36**	0	0	0
	**Northern Mountains**	4.9	8	9.09	**28.95**	0	2.44
	**Small Plain**	2.8	0	0	0	**30.77**	0
	**Western Hungary**	4.2	0	0	0	15.38	**46.34**
		**Great Plain**	**Transdanubian Hills**	**Transdanubian Mountains**	**Northern Mountains**	**Small Plain**	**Western Hungary**
**Validation 40.95%**	**Great Plain**	**76.62**	61.54	50	61.9	71.43	47.83
	**Transdanubian Hills**	6.49	**30.77**	16.67	4.76	0	0
	**Transdanubian Mountains**	0	0	**33.33**	4.76	0	0
	**Northern Mountains**	5.19	7.69	0	**28.57**	0	4.35
	**Small Plain**	5.19	0	0	0	**28.57**	0
	**Western Hungary**	6.49	0	0	0	0	**47.83**
