# Supplementary material for: Origin Identification of Hungarian Honey Using Melissopalynology, Physicochemical Analysis, and Near Infrared Spectroscopy
Source: Molecules. 2021 Nov 30;26(23):7274. doi: 10.3390/molecules26237274 (PMC8658813; doi:10.3390/molecules26237274)
Supplement: Supplementary file 1 [file molecules-26-07274-s001.zip › Table S4_mod.pdf]

Table S4 Confusion matrix of the model of the fused data for the classification of geographical origin

		Great Plain	Transdanubian Hills	Transdanubian Mountains	Northern Mountains	Small Plain	Western Hungary
**Traning 83.79%**	**Great Plain**	**94.67**	0	0	25.64	0	13.95
	**Transdanubian Hills**	0	**84**	0	0	0	0
	**Transdanubian Mountains**	0	0	**63.64**	0	0	0
	**Northern Mountains**	2	0	0	**74.36**	0	0
	**Small Plain**	3.33	0	0	0	**100**	0
	**Western Hungary**	0	16	36.36	0	0	**86.05**
		**Great Plain**	**Transdanubian Hills**	**Transdanubian Mountains**	**Northern Mountains**	**Small Plain**	**Western Hungary**
**Validation 81.60%**	**Great Plain**	**94.29**	0	0	25	0	14.29
	**Transdanubian Hills**	0	**84.62**	0	0	0	0
	**Transdanubian Mountains**	0	0	**50**	0	0	0
	**Northern Mountains**	2.86	0	0	**75**	0	0
	**Small Plain**	2.86	0	0	0	**100**	0
	**Western Hungary**	0	15.38	50	0	0	**85.71**
