# Supplementary material for: Origin Identification of Hungarian Honey Using Melissopalynology, Physicochemical Analysis, and Near Infrared Spectroscopy
Source: Molecules. 2021 Nov 30;26(23):7274. doi: 10.3390/molecules26237274 (PMC8658813; doi:10.3390/molecules26237274)
Supplement: Supplementary file 1 [file molecules-26-07274-s001.zip › Table S5_mod.pdf]

Table S5 List of the analyzed samples and their botanical and geographical sources

SampleID	Year	Longitude	Latitude	Altitude	District (*járás*)	County *(megye)*	Subregion	Region
Acacia_6	2015	21.75371	47.599	132	Debreceni járás	Hajdú-Bihar megye	Észak-Alföld	Great Plain
Acacia_29	2015	19.80056	48.0961	239	Salgótarjáni járás	Nógrád megye	Észak-Magyarország	Northern Mountains
Acacia_63	2015	20.43557	47.3364	84	Szolnoki járás	Jász-Nagykun-Szolnok megye	Észak-Alföld	Great Plain
Acacia_97	2016	20.4925	47.4995	87	Hevesi járás	Heves megye	Észak-Magyarország	Great Plain
Acacia_101	2016	20.3743665	47.8989887	169	Egri járás	Heves megye	Észak-Magyarország	Northern Mountains
Acacia_110	2018	21.5078944	47.6716617	121	Hajdúböszörményi járás	Hajdú-Bihar megye	Észak-Alföld	Great Plain
Acacia_134	2017	20.4925	47.4995	87	Hevesi járás	Heves megye	Észak-Magyarország	Great Plain
Acacia_145	2016	17.6165901	47.645238	114	Győri járás	Győr-Moson-Sopron megye	Nyugat-Dunántúl	Small Plain
Acacia_146	2017	17.6165901	47.645238	114	Győri járás	Győr-Moson-Sopron megye	Nyugat-Dunántúl	Small Plain
Acacia_148	2017	19.19536	47.8579	271	Rétsági járás	Nógrád megye	Észak-Magyarország	Northern Mountains
Acacia_153	2018	20.2015503	48.069769	214	NA	Heves megye	Észak-Magyarország	Northern Mountains
Acacia_157	2017	20.5	47	NA	NA	NA	NA	Great Plain
Acacia_160	2018	20.4925	47.4995	87	Hevesi járás	Heves megye	Észak-Magyarország	Great Plain
Acacia_171	2019	21.1347821	48.4657695	213	Encsi járás	Borsod-Abaúj-Zemplén megye	Észak-Magyarország	Northern Mountains
Acacia_172	2019	17.3015774	47.2744171	129	Pápai járás	Veszprém megye	Közép-Dunántúl	Small Plain
Acacia_173	2020	16.8894692	46.6713343	228	NA	Zala megye	Nyugat-Dunántúl	Western Hungary

SampleID	Year	Longitude	Latitude	Altitude	District *(járás)*	County *(megye)*	Subregion	Region
Acacia_178	2019	20.6560717	47.6435536	20.6560717	Füzesabonyi járás	Heves megye	Észak-Magyarország	Great Plain
Acacia_179	2017	19.6977	47.11838	130	Ceglédi járás	Pest megye	Közép-Magyarország	Great Plain
Acacia_182	2018	20.07364353	47.32830815	NA	Jászberényi járás	Jász-Nagykun-Szolnok megye	Észak-Alföld	Great Plain
Bastard indigo_66	2016	20.43557	47.3364	84	Szolnoki járás	Jász-Nagykun-Szolnok megye	Észak-Alföld	Great Plain
Bastard indigo_70	2016	20.6318292	47.3699089	91	Kunhegyesi járás	Jász-Nagykun-Szolnok megye	Észak-Alföld	Great Plain
Bastard indigo_72	2016	20.7546669	47.2162053	85	Karcagi járás	Jász-Nagykun-Szolnok megye	Észak-Alföld	Great Plain
Bastard indigo_131	2018	21.4279002	47.8452241	99	Hajdúnánási járás	Hajdú-Bihar megye	Észak-Alföld	Great Plain
Bastard indigo_152	2018	20.438295	47.3979955	86	NA	Jász-Nagykun-Szolnok megye	Észak-Alföld	Great Plain
Bastard indigo_177	2019	20.6560717	47.6435536	20.6560717	Füzesabonyi járás	Heves megye	Észak-Magyarország	Great Plain
Bastard indigo_194	2020	21.5373392	48.1728908	102	Ibrányi járás	Szabolcs-Szatmár-Bereg megye	Észak-Alföld	Great Plain
Chestnut_51	2015	17.5361	46.87973	206	Dunántúl	Vas megye	Nyugat-Dunántúl	Western Hungary
Chestnut_59	2015	16.8894692	46.6713343	228	NA	Zala megye	Nyugat-Dunántúl	Western Hungary
Chestnut_68	2016	16.5983457	47.6803063	224	Soproni járás	Győr-Moson-Sopron megye	Nyugat-Dunántúl	Western Hungary
Chestnut_73	2016	16.8894692	46.6713343	228	NA	Zala megye	Nyugat-Dunántúl	Western Hungary
Chestnut_105	2017	16.4938314	47.3455478	349	Kőszegi járás	Vas megye	Nyugat-Dunántúl	Western Hungary
Chestnut_128	2018	16.4938314	47.3455478	349	Kőszegi járás	Vas megye	Nyugat-Dunántúl	Western Hungary

SampleID	Year	Longitude	Latitude	Altitude	District (*járás*)	County *(megye)*	Subregion	Region
Chestnut_158	2016	17.88148	46.2031	193	Szigetvári járás	Baranya megye	Dél-Dunántúl	Transdanubian Hills
Chestnut_161	2019	16.8894692	46.6713343	228	NA	Zala megye	Nyugat-Dunántúl	Western Hungary
Chestnut_162	2019	17.77198	47.1672	NA	Veszprémi járás	Veszprém megye	Közép-Dunántúl	Transdanubian Mountains
Chestnut_186	2019	NA	NA	NA	NA	NA	NA	NA
Honeydew_48	2015	20.17316	47.5829	100	Jászapáti járás	Jász-Nagykun-Szolnok megye	Észak-Alföld	Great Plain
Honeydew_55	2015	19.95	47.8833	NA	Északi hegység	Északi hegység	Észak-Magyarország	Northern Mountains
Honeydew_62	2015	20.43557	47.3364	84	Szolnoki járás	Jász-Nagykun-Szolnok megye	Észak-Alföld	Great Plain
Honeydew_71	2016	NA	NA	NA	NA	NA	NA	NA
Honeydew_85	2016	21.3842	48.1865	96	Tokaji járás	Borsod-Abaúj-Zemplén megye	Észak-Magyarország	Northern Mountains
Honeydew_130	2018	21.626	47.5314	122	Debreceni járás	Hajdú-Bihar megye	Észak-Alföld	Great Plain
Honeydew_147	2017	19.5467411	47.2860696	138	Monori járás	Pest megye	Közép-Magyarország	Great Plain
Honeydew_170	2018	19.2893163	47.825568	300	Váci járás	Pest megye	Közép-Magyarország	Northern Mountains
Honeydew_188	2019	NA	NA	NA	NA	NA	NA	NA
Honeydew_189	2020	NA	NA	NA	NA	NA	NA	NA
Linden_35	2015	17.88148	46.2031	193	Szigetvári járás	Baranya megye	Dél-Dunántúl	Transdanubian Hills
Linden_60	2015	20.43557	47.3364	84	Szolnoki járás	Jász-Nagykun-Szolnok megye	Észak-Alföld	Great Plain
Linden_103	2016	19.7977516	47.1716447	106	Ceglédi járás	Pest megye	Közép-Magyarország	Great Plain
Linden_129	2018	17.88148	46.2031	193	Szigetvári járás	Baranya megye	Dél-Dunántúl	Transdanubian Hills
Linden_136	2017	18.0237865	46.7892822	154	Tabi járás	Somogy megye	Dél-Dunántúl	Transdanubian Hills
Linden_149	2019	17.88148	46.2031	193	Szigetvári járás	Baranya megye	Dél-Dunántúl	Transdanubian Hills
Linden_150	2017	18.9705776	47.7848912	250	NA	NA	NA	NA

SampleID	Year	Longitude	Latitude	Altitude	District (*járás*)	County *(megye)*	Subregion	Region
Linden_156	2017	20.5	47	NA	NA	NA	NA	Great Plain
Linden_164	2019	17.77198	47.1672	NA	Veszprémi járás	Veszprém megye	Közép-Dunántúl	Transdanubian Mountains
Linden_165	2018	17.88148	46.2031	193	Szigetvári járás	Baranya megye	Dél-Dunántúl	Transdanubian Hills
Linden_183	2019	17.77198	47.1672	NA	Veszprémi járás	Veszprém megye	Közép-Dunántúl	Transdanubian Mountains
Rape_88	2016	20.4925	47.4995	87	Szolnoki járás	Jász-Nagykun-Szolnok megye	Észak-Alföld	Great Plain
Rape_133	2018	21.2366013	48.030817	94	Győri járás	Győr-Moson-Sopron megye	Nyugat-Dunántúl	Small Plain
Rape_155	2019	NA	NA	NA	Győri járás	Győr-Moson-Sopron megye	Nyugat-Dunántúl	Small Plain
Rape_168	2017	20.74598	48.3108	132	Győri járás	Győr-Moson-Sopron megye	Nyugat-Dunántúl	Small Plain
Rape_169	2020	16.5406843	47.3894949	277	Békési járás	Békés megye	Dél-Alföld	Great Plain
Rape_174	2020	20.4925	47.4995	87	Paksi járás	Tolna megye	Dél-Dunántúl	Great Plain
Rape_176	2019	20.6560717	47.6435536	20.6560717	NA	Jász-Nagykun-Szolnok megye	Észak-Alföld	Great Plain
Rape_180	2016	20.4925	47.4995	87	Balatonalmádi járás	Veszprém megye	Közép-Dunántúl	Great Plain
Rape_181	2017	20.4925	47.4995	87	Edelényi járás	Borsod-Abaúj-Zemplén megye	Észak-Magyarország	Northern Mountains
Rape_184	2020	18.7576092	47.1385777	0	Füzesabonyi Járás	Heves megye	Észak-Magyarország	Great Plain
Milkweed_32	2015	19.476022	46.7514033	108	Hevesi járás	Heves megye	Észak-Magyarország	Great Plain
Milkweed_50	2015	19.6821	46.6889	111	Tiszavasvári járás	Szabolcs-Szatmár-Bereg megye	Észak-Alföld	Great Plain
Milkweed_52	2015	19.2313617	47.3011895	110	NA	NA	NA	NA
Milkweed_132	2018	19.4833076	46.4278497	126	Edelényi járás	Borsod-Abaúj-Zemplén megye	Észak-Magyarország	Northern Mountains

SampleID	Year	Longitude	Latitude	Altitude	District (*járás*)	County *(megye)*	Subregion	Region
Milkweed_185	2019	18.9822596	46.2519797	95	Kőszegi járás	Vas megye	Nyugat-Dunántúl	Western Hungary
Milkweed_187	2020	NA	NA	NA	Hevesi járás	Heves megye	Észak-Magyarország	Great Plain
Milkweed_190	2020	19.2225016	46.8752316	99	Füzesabonyi Járás	Heves megye	Észak-Magyarország	Great Plain
Milkweed_191	2020	19.6928133	46.9080769	122	Hevesi járás	Heves megye	Észak-Magyarország	Great Plain
Milkweed_192	2020	19.4326126	47.2995594	128	Hevesi járás	Heves megye	Észak-Magyarország	Great Plain
Milkweed_193	2020	20.6052	48.519135	372	NA	Fejér megye	Közép-Dunántúl	Great Plain
Sunflower_65	2015	20.43557	47.3364	84	Kecskeméti járás	Bács-Kiskun megye	Dél-Alföld	Great Plain
Sunflower_137	2015	17.6165901	47.645238	114	Kiskunfélegyházi járás	Bács-Kiskun megye	Dél-Alföld	Great Plain
Sunflower_138	2016	17.6165901	47.645238	114	Gyáli járás	Pest megye	Közép-Magyarország	Great Plain
Sunflower_139	2017	17.6165901	47.645238	114	Kiskunhalasi járás	Bács-Kiskun megye	Dél-Alföld	Great Plain
Sunflower_140	2017	21.2126944	46.8121382	84	NA	Bács-Kiskun megye	Dél-Alföld	Great Plain
Sunflower_141	2017	18.8119	46.53001	100	NA	NA	NA	NA
Sunflower_151	2018	20.59977	47.476	87	Kunszentmiklósi járás	Bács-Kiskun megye	Dél-Alföld	Great Plain
Sunflower_166	2019	18.2076416	47.40838766	233	Kecskeméti járás	Bács-Kiskun megye	Dél-Alföld	Great Plain
Sunflower_167	2017	20.74598	48.3108	132	Monori járás	Pest megye	Közép-Magyarország	Great Plain
Sunflower_175	2017	20.6560717	47.6435536	20.6560717	Edelényi járás	Borsod-Abaúj-Zemplén megye	Észak-Magyarország	Northern Mountains

NA – Not available
