# Supplementary material for: Origin Identification of Hungarian Honey Using Melissopalynology, Physicochemical Analysis, and Near Infrared Spectroscopy
Source: Molecules. 2021 Nov 30;26(23):7274. doi: 10.3390/molecules26237274 (PMC8658813; doi:10.3390/molecules26237274)
Supplement: Supplementary file 1 [file molecules-26-07274-s001.zip › Table S6_mod.pdf]

Table S6 - Pretreatments of the NIR spectra

Number	Pretreatment (combination)
1	Savitzky-Golay Smoothing 13 sample points, 2nd polynomial no derivation
2	Savitzky-Golay Smoothing 17 sample points, 2nd polynomial no derivation
3	Savitzky-Golay Smoothing 21 sample points, 2nd polynomial no derivation
4	Standard Normal Variate
5	Multiplicative scatter correction
6	Detrending
7	Detrending + Multiplicative scatter correction
8	Detrending + Standard Normal Variate
9	Savitzky-Golay Smoothing 13 sample points, 2nd polynomial no derivation + Standard Normal Variate
10	Savitzky-Golay Smoothing 17 sample points, 2nd polynomial no derivation + Standard Normal Variate
11	Savitzky-Golay Smoothing 21 sample points, 2nd polynomial no derivation + Standard Normal Variate
12	Savitzky-Golay Smoothing 13 sample points, 2nd polynomial no derivation + Multiplicative scatter correction
13	Savitzky-Golay Smoothing 17 sample points, 2nd polynomial no derivation + Multiplicative scatter correction
14	Savitzky-Golay Smoothing 21 sample points, 2nd polynomial no derivation + Multiplicative scatter correction
15	Savitzky-Golay Smoothing 13 sample points, 2nd polynomial no derivation + Detrending
16	Savitzky-Golay Smoothing 17 sample points, 2nd polynomial no derivation + Detrending
17	Savitzky-Golay Smoothing 21 sample points, 2nd polynomial no derivation + Detrending
18	Savitzky-Golay Smoothing 13 sample points, 2nd polynomial no derivation + Detrending + Standard Normal Variate
19	Savitzky-Golay Smoothing 17 sample points, 2nd polynomial no derivation + Detrending + Standard Normal Variate
20	Savitzky-Golay Smoothing 21 sample points, 2nd polynomial no derivation + Detrending + Standard Normal Variate
21	Savitzky-Golay Smoothing 13 sample points, 2nd polynomial no derivation + Detrending + Multiplicative scatter correction
22	Savitzky-Golay Smoothing 17 sample points, 2nd polynomial no derivation + Detrending + Multiplicative scatter correction
23	Savitzky-Golay Smoothing 21 sample points, 2nd polynomial no derivation + Detrending + Multiplicative scatter correction
24	Savitzky-Golay Smoothing 21 sample points, 2nd polynomial no derivation + Savitzky-Golay Smoothing 21 sample points, 2nd polynomial 1st derivative
25	Savitzky-Golay Smoothing 21 sample points, 2nd polynomial no derivation + Savitzky-Golay Smoothing 21 sample points, 2nd polynomial 2nd derivative
26	Savitzky-Golay Smoothing 21 sample points, 2nd polynomial no derivation + Savitzky-Golay Smoothing 13 sample points, 2nd polynomial 1st derivative

Number	Pretreatment (combination)
27	Savitzky-Golay Smoothing 21 sample points, 2nd polynomial no derivation + Savitzky-Golay Smoothing 13 sample points, 2nd polynomial 2nd derivative
28	Savitzky-Golay Smoothing 21 sample points, 2nd polynomial no derivation + Savitzky-Golay Smoothing 17 sample points, 2nd polynomial 1st derivative
29	Savitzky-Golay Smoothing 21 sample points, 2nd polynomial no derivation + Savitzky-Golay Smoothing 17 sample points, 2nd polynomial 2nd derivative
30	Savitzky-Golay Smoothing 13 sample points, 2nd polynomial no derivation + Savitzky-Golay Smoothing 21 sample points, 2nd polynomial 1st derivative
31	Savitzky-Golay Smoothing 13 sample points, 2nd polynomial no derivation + Savitzky-Golay Smoothing 21 sample points, 2nd polynomial 2nd derivative
32	Savitzky-Golay Smoothing 13 sample points, 2nd polynomial no derivation + Savitzky-Golay Smoothing 13 sample points, 2nd polynomial 1st derivative
33	Savitzky-Golay Smoothing 13 sample points, 2nd polynomial no derivation + Savitzky-Golay Smoothing 13 sample points, 2nd polynomial 2nd derivative
34	Savitzky-Golay Smoothing 13 sample points, 2nd polynomial no derivation + Savitzky-Golay Smoothing 17 sample points, 2nd polynomial 1st derivative
35	Savitzky-Golay Smoothing 13 sample points, 2nd polynomial no derivation + Savitzky-Golay Smoothing 17 sample points, 2nd polynomial 2nd derivative
36	Savitzky-Golay Smoothing 17 sample points, 2nd polynomial no derivation + Savitzky-Golay Smoothing 21 sample points, 2nd polynomial 1st derivative
37	Savitzky-Golay Smoothing 17 sample points, 2nd polynomial no derivation + Savitzky-Golay Smoothing 21 sample points, 2nd polynomial 2nd derivative
38	Savitzky-Golay Smoothing 17 sample points, 2nd polynomial no derivation + Savitzky-Golay Smoothing 17 sample points, 2nd polynomial 1st derivative
39	Savitzky-Golay Smoothing 17 sample points, 2nd polynomial no derivation + Savitzky-Golay Smoothing 17 sample points, 2nd polynomial 2nd derivative
40	Savitzky-Golay Smoothing 17 sample points, 2nd polynomial no derivation + Savitzky-Golay Smoothing 13 sample points, 2nd polynomial 1st derivative
41	Savitzky-Golay Smoothing 17 sample points, 2nd polynomial no derivation + Savitzky-Golay Smoothing 13 sample points, 2nd polynomial 2nd derivative
